# Bacteria Modulate the CD8+ T Cell Epitope Repertoire of Host Cytosol-Exposed Proteins to Manipulate the Host Immune Response

**DOI:** 10.1371/journal.pcbi.1002220

**Published:** 2011-10-13

**Authors:** Yaakov Maman, Ran Nir-Paz, Yoram Louzoun

**Affiliations:** 1 Department of Mathematics and Gonda Brain Research Center, Bar-Ilan University, Ramat Gan, Israel; 2 Department of Clinical Microbiology and Infectious Diseases, Hadassah-Hebrew University Medical Center, Jerusalem, Israel; La Jolla Institute for Allergy and Immunology, United States of America

## Abstract

The main adaptive immune response to bacteria is mediated by B cells and CD4+ T-cells. However, some bacterial proteins reach the cytosol of host cells and are exposed to the host CD8+ T-cells response. Both gram-negative and gram-positive bacteria can translocate proteins to the cytosol through type III and IV secretion and ESX-1 systems, respectively. The translocated proteins are often essential for the bacterium survival. Once injected, these proteins can be degraded and presented on MHC-I molecules to CD8+ T-cells. The CD8+ T-cells, in turn, can induce cell death and destroy the bacteria's habitat. In viruses, escape mutations arise to avoid this detection. The accumulation of escape mutations in bacteria has never been systematically studied. We show for the first time that such mutations are systematically present in most bacteria tested. We combine multiple bioinformatic algorithms to compute CD8+ T-cell epitope libraries of bacteria with secretion systems that translocate proteins to the host cytosol. In all bacteria tested, proteins not translocated to the cytosol show no escape mutations in their CD8+ T-cell epitopes. However, proteins translocated to the cytosol show clear escape mutations and have low epitope densities for most tested HLA alleles. The low epitope densities suggest that bacteria, like viruses, are evolutionarily selected to ensure their survival in the presence of CD8+ T-cells. In contrast with most other translocated proteins examined, *Pseudomonas aeruginosa'*s ExoU, which ultimately induces host cell death, was found to have high epitope density. This finding suggests a novel mechanism for the manipulation of CD8+ T-cells by pathogens. The ExoU effector may have evolved to maintain high epitope density enabling it to efficiently induce CD8+ T-cell mediated cell death. These results were tested using multiple epitope prediction algorithms, and were found to be consistent for most proteins tested.

## Author Summary

Bacterial proteins are mainly exposed to B-cells and CD4+ T-cells, while CD8+ T-cells (CTL) typically respond to viruses. The limitation of the CTL response to viruses results from processing pathways of epitopes presented to CTLs. These epitopes usually stem from proteins expressed in the cytosol. Such proteins are eventually degraded and presented on MHC-I molecules to CTLs. However bacterial Type III secretion system (T3SS) effectors also have an access to the host cytosol and may also be exposed to CTL response. Thus, we can assume that this group of proteins undergoes selection against the presentation of CTL epitopes, as seen in viral proteins. Using multiple epitope prediction algorithms, we show that most T3SS effectors, as well as LLO, and ActA in *Listeria monocytogenes* and ESAT-6 proteins in *Mycobacterium tuberculosis*, are systematically selected to reduce the number and quality of their epitopes. The exception in this respect is the *Pseudomonas aeruginosa* effector ExoU that has high density of high quality epitopes. Since ExoU is known to induce rapid cell death in hosts cells, we assume that *P.aeruginosa* utilize the immune response to induce such death. The *E.coli* epitope density is highly variable among strains.

## Introduction

CD8+ T-cells recognize mainly cytosolic epitopes presented on MHC-I molecules. Their response is thus assumed to be directed mainly against viruses [1], [2], [3]. Bacterial proteins, on the other hand, are typically expressed outside the cytosol, and as such, induce CD4+ T-cells and B cells responses [4], [5], [6], [7], [8], [9], [10], and are not expected to induce a CTL response in the classical pathway. For such a response to occur, these proteins must reach MHC-I proteins in the ER.

One extensively studied possible mechanism for the presentation of bacterial epitope is "cross presentation". In general, "cross presentation" refers to the transfer of peptides from the MHC-II presentation pathway to the MHC-I presentation pathway and vice versa [11], [12], [13], [14]. Specifically, peptides of intracellular bacterial proteins derived from endosomal cleavage are presented on MHC-I molecules. This could take place in two ways: either the peptides are translocated to the cytosol, cleaved by the proteasome and delivered to the ER through TAP where they bind to MHC class I molecules, or endosomal peptides bind to MHC-I molecules probably in the endocytic compartment itself (for a review see [15]).

Another much more direct mechanism is the translocation of bacterial protein to the cytosol by highly conserved secretion systems. Such systems exist in a variety of bacteria. The secretion system that has been most characterized is the type III secretion system (T3SS) in gram-negative bacteria. The T3SS is a complex that allows bacteria to deliver protein effectors across eukaryotic cellular membranes through needle-like structure. In the cytosol, T3SS effectors exert many effects, such as cellular invasion [16], modulation of host immune response [17], [18] and apoptosis [19]. Another secretion system is the ESX-1 system in *Mycobacterium tuberculosis* (TB) [20]. Similar systems (also called ESX/T7S systems) exist in other gram positive bacteria as well [21]. However, since these systems do not have a needle-like structure, they cannot inject proteins through the plasma membrane. Nevertheless, TB is an intracellular bacterium and its secreted proteins can gain access to the cytosol [22]. The third characterized system that injects cytosolic proteins was studied in the intracellular cytosolic bacterium *Listreria monocytogenes* that injects the virulence factors Listeriolysin O (LLO) and ActA to the host cytosol [23].

These proteins are good candidates for presentation on MHC class I molecules. In similar situations, viruses avoid the presentation of CD8+ T cell epitopes through escape mutations [1], [24], [25], [26], [27], [28]. Here we study bacterial sequences to test whether bacteria adopt a similar strategy of epitope removal. Specifically, we systematically compute the epitope density in bacterial effector proteins and show a clear selection against the presentation of epitopes. This selection is highly specific to cytosolic proteins.

Evidences for MHC-I presentation in bacteria are limited to specific bacterial proteins, such as the *L*. *monocytogenes* proteins listeriolysin O [29] and ActA [30], the *Bordetella* pertussis adenylatecyclase toxin [31], the TB CFP10 antigen [22] and *Streptococci* protein streptolysin O [32]. A CTL response to extracellular pathogens was also suggested by some studies. Bergman et al. showed that the CTL response has a critical role in eliminating *Yersinia* infections, and that this response is directed against Yops, the secreted effector proteins of *Yersinia* [33]. Other studies about the CTL response against extracellular pathogen were carried out by Meissner et al. that demonstrated a vigorous CD8+ T cell influx into the lung in response to *Pneumocystis*, an extracellular fungal pathogen [34], and by Mehrzad et al. (2008) that showed that trafficking of CD8+ T cells during initiation of *Escherichia coli* mastitis is accelerated when increasing the *E. coli* inoculum dose [35]. However, none of these studies suggested the existence of escape mutations in bacteria. We here show that such escape mutations are common in most tested effector proteins.

The field of immunomics has made a significant leap forward in the last decades. Tools for epitope prediction have been developed for most branches of the immune system. The precision of CD8+ T cell epitopes prediction processing and presentation tools has reached the level that allows a systematic prediction of full organism epitope libraries. CTL epitopes are typically 8-10 amino acid long peptides, bound to MHC-I molecules [36]. These peptides are presented after proteasomal cleavage and transfer from the cytosol via TAP to the ER [37], [38], where they bind to MHC-I molecules. We have developed a precise cleavage prediction algorithm [39] and used TAP [40] and MHC binding [41] algorithms, which were found to be precise enough for most MHC-I alleles, to compute presented epitopes densities [24], [25], [42], [43], [44]. The precision of these densities has been tested in depth [24], [43], [44].

In this study, we study the epitope density of proteins in a group of representative bacteria expressing proteins translocated to the cytosol. Three of them, *Escherichia coli*, *Shigella flexneri*, and *Pseudomonas aureginosa*, are gram-negative T3SS-containing bacteria. In parallel, we study cytosol-exposed proteins from the gram-positive *Listeria monocytogenes* and *Mycobacterium tuberculosis.* In order to validate the results, we repeat the analysis using three different algorithms to test that the results obtained are not the artifact of the specific MHC binding algorithms used.

## Results

### *SIR* score

**Figure 1:**
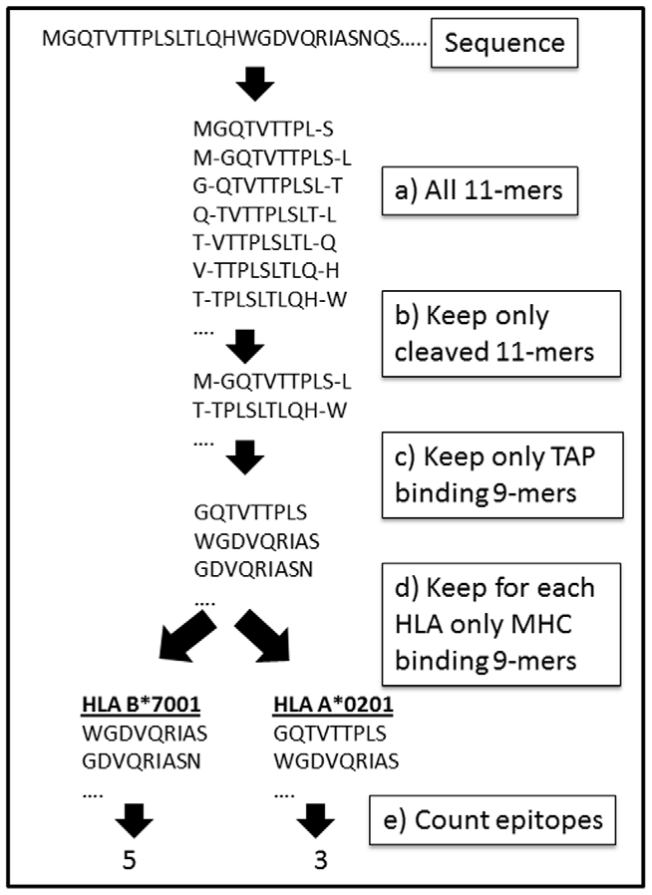
Algorithm for epitope prediction and *SIR* score computation. Each protein is divided into all nine-mers and the appropriate flanking positions (a). For each eleven-mer (a nine-mer and the C and N flanking positions), a cleavage score is computed (b). We compute for all peptides with a positive cleavage score a TAP binding score and choose only supra-threshold peptides (c). The MHC binding score of all TAP binding and cleaved nine-mers is computed (d). Nine-mers passing all these stages are defined as epitopes. We then compute the number of epitopes per protein per HLA allele (e). The ratio between the number of predicted epitopes and the parallel number on a random sequence with a random amino acid distribution is defined as the SIR score.

We have previously conducted a systematic analysis of the predicted CTL epitope repertoire in human and foreign proteins, and defined the normalized epitope density of a protein or an organism as the Size of Immune Repertoire (*SIR*) score [24], [25], [39], [43], [45], [46], [47]. The number of predicted CTL epitopes from a sequence was computed by applying a sliding window of nine amino acids, and computing for each nine-mer and its two flanking residues whether it is cleaved by the proteasome and whether it binds to TAP channels and to a given MHC-I allele (Figure 1). The *SIR* score was defined as the ratio between the computed CTL epitope density (fraction of nine-mers that were predicted to be epitopes) and the epitope density expected within the same number of random nine-mers. The choice of the “random” nine-mers will be discussed in the following section. An average *SIR* score of less than 1 represents an under-presentation of epitopes, whereas an average *SIR* score of more than 1 represents an over-presentation of epitopes. For example, assume a hypothetical sequence of 1,008 amino acids (1,000 nine-mers) containing 15 HLA A*0201 predicted epitopes. If the average epitope density of HLA A*0201 in a large number of random proteins was 0.01 (i.e. 10 epitopes in 1,000 nine-mers), then the *SIR* score of the sequence for HLA A*0201 would be 1.5 (15/10). The average *SIR* score of a protein was defined as the average of the *SIR* scores for each HLA allele studied, weighted by the allele's frequency in the average human population. These results obviously depend on the definition of a “random protein”. We have thus tested multiple such definitions.

### Defining the baseline for the *SIR* score

An important issue in the analysis of selection is the baseline against which the number of epitopes of a given protein is compared. In previous work on viruses [24], [25], [43], [48], we have compared human to parallel non-human viruses as a negative control. However, bacteria have a wide range of possible hosts and purely non-human homolog bacteria often do not exist. We thus use three different background distributions to compare with:

- In order to produce the first baseline, we have produced a long random sequence of amino acids that have the typical amino acid sequence composition of viral proteins. We have then computed the epitope density in this sequence for each allele, and defined it as the average expected epitope density for this allele. This value is used as the denominator of the *SIR* score for each allele. The advantage of this baseline is that it is uniform over all proteins.
- A second, baseline, which is protein dependent, is the average epitope density over 50 sequences produced by permuting the order of the amino acids in the protein (scrambled versions of the protein). Although these scrambled versions have no biological viability, they gives a picture of the typical epitope density of proteins with similar amino acid distribution in which selection does not play a role. Such a baseline can, for example, neutralize the effect of hydrophobicity on the epitope density [48], [49], [50], [51].
- The third baseline is the epitope density in a randomly selected group of proteins in the same bacteria. Such a baseline represents the difference between protein groups (the random group and the group of interest) in the same bacteria.

In the following section, we will use all three baselines to show that selection occurs in cytosolic bacterial proteins.

### Typical bacterial proteins show no specific escape mutations

While most viral proteins are expressed in the cytosol and exposed to the MHC-I presentation pathway, bacterial proteins are usually expressed either within the bacteria or within the endosome of a phagocyte, and hence are not exposed to the MHC-I presentation pathway. Thus, we expected that in contrast with viruses [24], [25], [42], [43], [44], the *SIR* score of all bacterial proteins would be distributed around 1.

**Figure 2:**
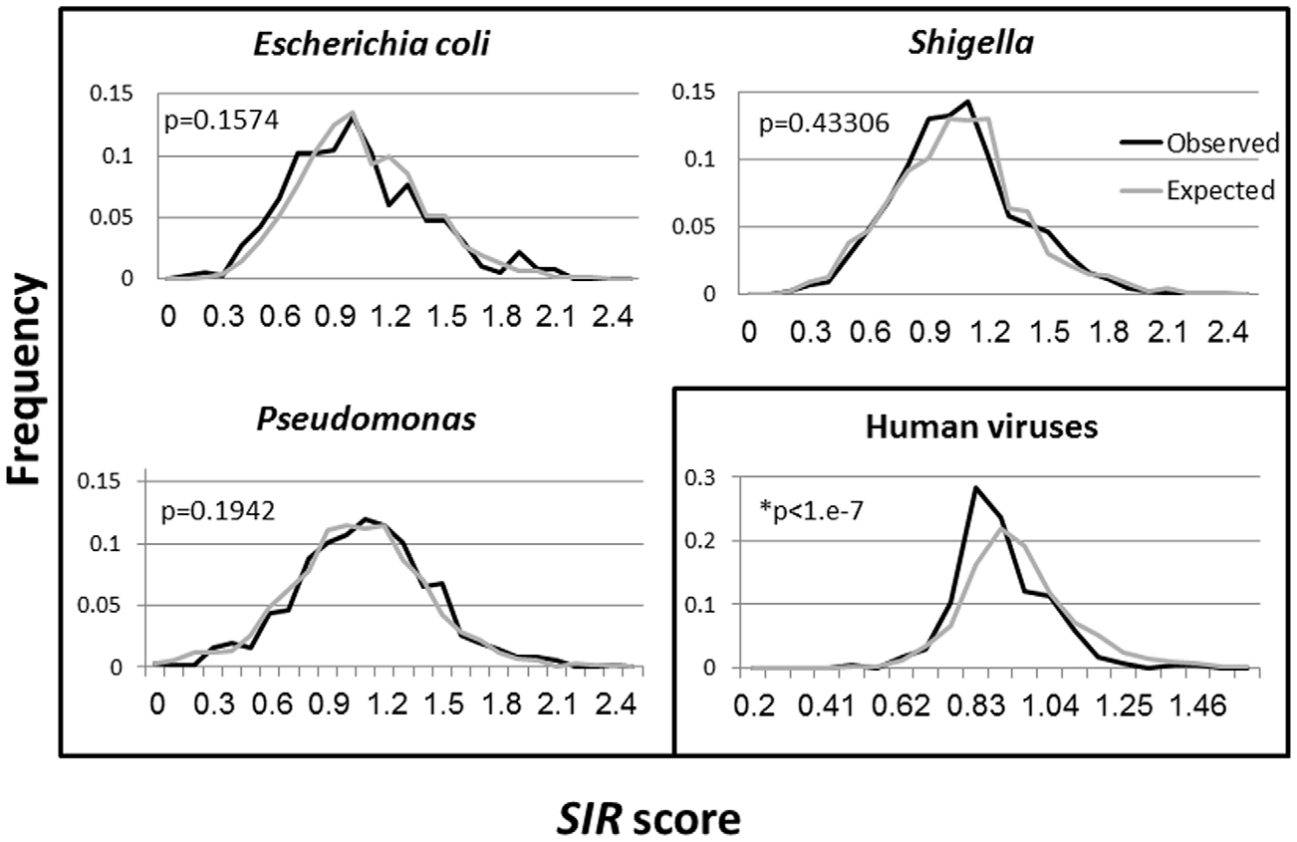
Upper row and lower left drawings - Histograms of SIR score values for real and scrambled sequences of bacterial proteins. The x axis is the SIR scores and the y axis is the frequency of sequence with such an SIR score. Each drawing represents a different bacterium. The dark lines are the real sequences and the gray lines are the values obtained for scrambled versions of the same sequences. The distributions of the real and scrambled sequences overlap showing that bacteria, unlike viruses, do not generally accumulate escape mutations in their CTL-epitopes (P = 0.4306,0.1574 and 0.1942 for *Shigella*, *E.coli* and *Pseudomonas*, respectively). The lower left drawing is the SIR score histogram in human viruses (dark line) and the parallel in non human viruses (gray line). The average human virus SIR score is lower than the one of non-human viruses, revealing the accumulation of escape mutations in human viral proteins (P value<1.e-7).

The epitope density of a protein is affected by two main elements: A) a direct negative selection of epitopes through the immune response against pathogens expressing proteins carrying many epitopes, B) inherent features of the protein, determining its amino acid usage, which in turn affects the epitope density. In order to check for the direct effect of negative selection, we compared the *SIR* score of each protein, not only to 1, but also to the *SIR* score of scrambled sequences with identical amino acid distribution (that we denote as the neutral *SIR* score). When all bacterial proteins are analyzed, the *SIR* scores distributions of the real and scrambled proteins are similar and are close to 1 (Figure 2, T-test , P-value>0.15 for all bacteria tested).

### Bacterial proteins translocated to the cytosol are selected to evade CTL recognition

While most bacterial proteins have the expected epitope density (Figure 2), the epitope density of bacterial proteins that are secreted to the host cytosol may be affected by CTL mediated selection. Such proteins are often present at high concentrations in the cytosol and are exposed to the MHC-I presentation pathway.

Five examples of such bacteria are tested in this study: *P. aeruginosa, S.flexneri, E.coli, L.monocytogenes* and *M.tuberculosis*. Before examining each bacterium separately, we compared the *SIR* score in all cytosolic proteins of these bacteria against the *SIR* score in randomly selected proteins, against scrambled versions of themselves and against 1. In all three cases, the *SIR* score of the cytosolic bacterial proteins were significantly lower (ANOVA P-value<1.e-11 for all three tests). No significant differences were found between *the SIR* score of randomly selected proteins and their scrambled versions. (ANOVA P-value = 0.9114). These results suggest that bacterial proteins located in the host cytosol have evolved to evade CTL recognition.

The most characterized bacterial cytosolic proteins are the effector proteins of secretion systems in gram negative bacteria. We analyzed the *SIR* score of bacterial proteins in bacteria where we had a clear definition of effector proteins. We first analyzed *P. aeruginosa* , *S. flexneri* and *E.coli* as models for gram negative bacteria with Type III secretion systems. *S. flexneri* represents intracellular bacteria, *P. aeruginosa* represents cytopathic extracellular bacteria *and E. coli* (entropathogenic (EPEC) and enterohemoreagic (EHEC) subgroups) represent extracellular non-cytopathic bacteria. In the following sections, we show that systematically, in most bacteria tested, the epitope density in effector proteins is lower than expected, with one interesting exception.

### *Pseudomonas aeruginosa* effectors

*P. aeruginosa* is a major cause of health care associated infections. It has only four known effector proteins: ExoS, ExoT, ExoU and ExoY. Almost all *Pseudomonas* strains contain ExoY and ExoT (89% and 96%, respectively) [52]. However, nearly all strains have either the ExoS or the ExoU gene but not both [53]. ExoS has several adverse effects on the host cell, including actin cytoskeleton disruption (associated with cell rounding) and inhibition of DNA synthesis, vesicular trafficking, endocytosis and cell death. ExoS induced stress is characterized by slow death induction of the infected cell.

ExoU is a potent phospholipase that is capable of causing rapid cell death in eukaryotic cells. ExoU containing strains of *P. aeruginosa* are much more cytopathic than their ExoS containing counterparts, which are more invasive.

#### *In* Pseudomonas aeruginosa - *all effectors proteins have low epitope densities, except for the ones inducing cell death*

**Figure 3:**
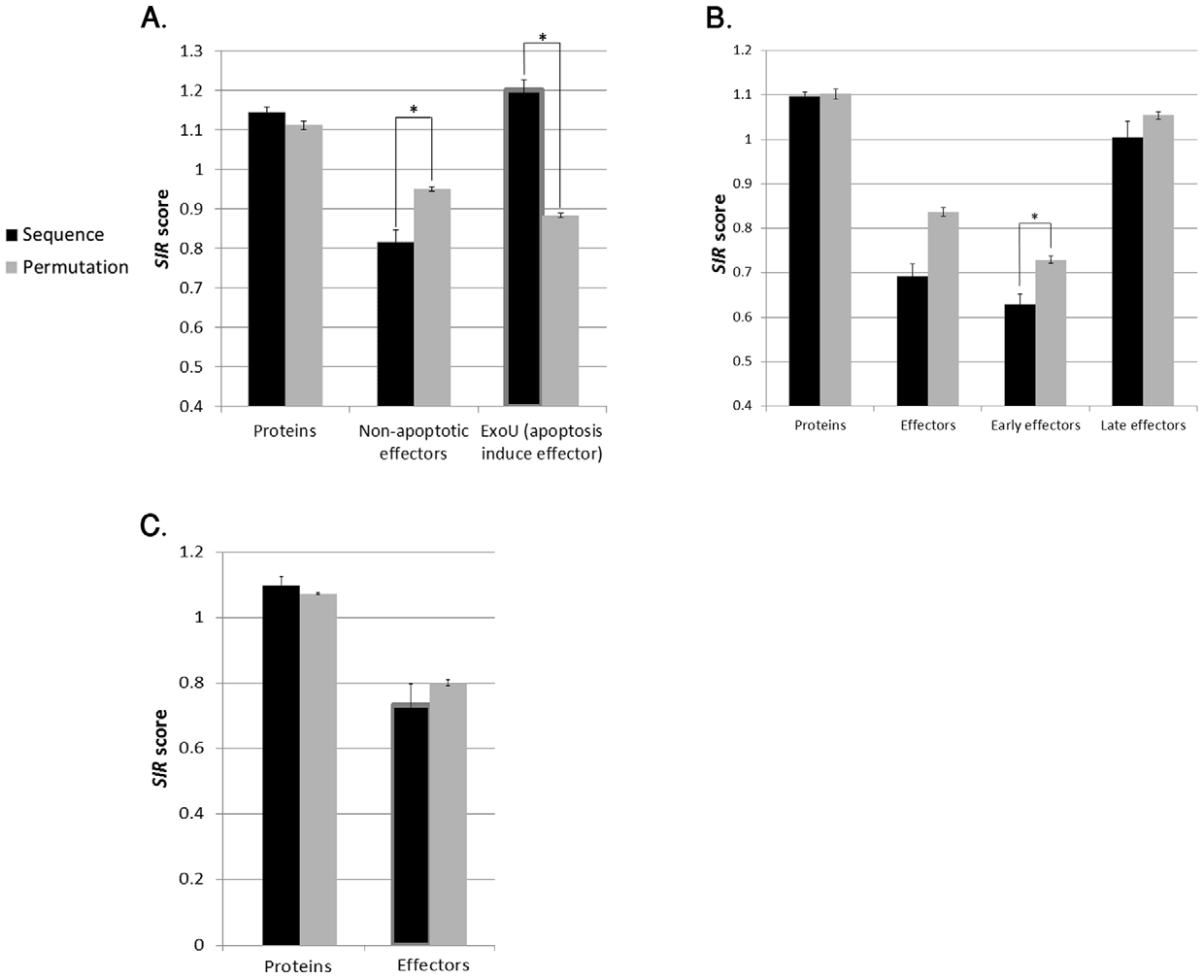
*SIR* score of T3SS effectors and non-effectors proteins in *Shigella flexneri*, *Pseudomonas aeruginosa* and *Escherichia coli*. A) Comparison between the average SIR score in real sequences and in scrambled sequences in *Pseudomonas aureginosa*. The first column is the average over 400 randomly selected proteins and the second column is the average of ExoT, ExoS and ExoY (non-necrotic effectors). The third column is the average of the necrotic effector ExoU. The first column has a similar average for the real and scrambled sequences (P value>0.18). In the second column, the real T3SS effectors sequences have a lower averaged *SIR* score than randomly selected proteins and also than expected from their scrambled sequences (P values = 2.4e-6 and 0.011, respectively). The third column demonstrates that ExoU has a higher SIR score than randomly selected proteins and than expected from its scrambled sequences (P-value<1e-4). B) Comparison between the average *SIR* score in real sequences and in scrambled sequences in *Shigella flexneri*. The first column is the average over 400 randomly selected proteins. The second column is the average over all T3SS effectors. The third and fourth columns are the averages over all early secreted and late secreted effectors, respectively. The first column has a similar average for the real and scrambled sequences (P value>0.3). Again, as in *P. aureginosa*, effectors have a lower *SIR* score average than randomly selected proteins, and also than expected from their scrambled sequences (second, third and fourth columns). Moreover, this bias is much stronger in early secreted effectors (*P-value<8e-4.). The differences in the second and fourth columns (overall and late effectors) are not significant (P-value>0.2). C) Comparison between the average *SIR* score in real sequences and in scrambled sequences in *Escherichia coli*. The left column is the average over 400 randomly selected proteins and the right column is the average over all T3SS effectors. The left column has a similar average for the real and scrambled sequences (P value>0.15). In the right columns, the real T3SS effectors sequences have a lower SIR score average than randomly selected proteins (P-value = 2.4e-3). Although *E.coli* effectors have a lower averaged *SIR* score than expected from their scrambled sequences, the difference is not significant (P-value>0.3). Bordered bars represent results that are not consistent with the other MHC-I binding algorithms, MLVO and NetMHC (figures 6 and 7, respectively).

As shown in Figure 3A, in all *P. aeruginosa* effectors besides ExoU, the *SIR* score was significantly lower than 1 (T-test P-value<1.e-4), lower than the neutral *SIR* score (the *SIR* score of scrambled versions of the same proteins) (ANOVA P-value = 0.011), and lower than the *SIR* scores of randomly selected non-effector proteins (ANOVA P-value = 2.4e-6). All randomly selected -non-effector- proteins showed no difference between the actual and the neutral *SIR* score. These results suggest that *P. aeruginosa* effectors, ExoS, ExoY and ExoT, are selected to present less CTL-epitopes. The special case of the fourth effector, ExoU will be discussed later. *SIR* scores for each effector separately are given in Supplementary Material, table S2.

#### Shigella flexneri *effectors*

*Shigella* species are gram-negative bacteria that can colonize the intestinal epithelium by exploiting epithelial-cell functions [54]. The first step of the *Shigella* infection is crossing the intestinal epithelial barrier. When this is achieved, the bacterium enters the macrophages that reside within the microfold-cell (M-cell) pocket. *S.flexneri* effectors can be divided into early and late subsets. The early effectors, IpaA, IpaB, IpaC, IpgB1, IpgB2, IpgD and VirA, are secreted early in the infection, immediately after contact with the epithelial cell. Their function is mainly to promote bacterial basolateral entry into polarized epithelial cells.

When *Shigella* reaches the epithelium, it secretes the late effectors subset: IcsB, VirA, OspF, OspG and IpaH family proteins (VirA is secreted in both stages). These effectors facilitate bacteria intracellular survival, promote intra and intercellular movement, and modulate the host inflammatory response [55].

#### *In* Shigella flexneri, *all effectors have a low epitope density, but early expression effectors have the lowest epitope density*

We have previously shown in viruses that early expressed proteins are under a more stringent pressure than late ones [24], [25], [43]. We have tested whether the same phenomenon occurs in bacteria. In Figure 3B, the *SIR* score of early and late effectors as well as non-effectors proteins is compared to their scrambled versions. Again, in non-effectors proteins, the neutral *SIR* score is very similar to the actual *SIR* score. In effectors, the *SIR* score is significantly lower than both 1 (T-test P-value<1.e-15) and than other bacterial proteins in the *Shigella* (ANOVA P-value = 4.8e-4). The decrease against the neutral score is not significant (ANOVA P-value = 0.294). However, when using other prediction algorithm, this decrease is also significant, as shall be further discussed.

The decrease in the epitope number was much more significant in early effectors than in late effectors (ANOVA P-value 1.0e-10). Early effectors have a significantly lower *SIR* score than 1 (T-Test P-value = 6.4e-19), other proteins (ANOVA P-value = 3.16e-13), or their own Neutral *SIR* score (ANOVA P-value 8.8e-4). In late effectors, the decrease was not significant (P-values = 0.69, 0.12 and 0.71 is the three tests above), as was observed in viruses [24], [25], [43].

Therefore, beyond the general CTL-induced selection observed in effectors, these results suggest a differential force of selection in *S. flexneri* effectors proteins, where early effectors are under stronger pressure to hide their CD8+ T cell epitopes than late ones.

#### Escherichia coli *effectors*

*Escherichia coli* are gram negative bacteria whose main natural habitat is the gastrointestinal tract of warm-blooded organisms (for review see [56]). Most strains exist as harmless symbionts, but some are pathogenic. Two of them, EPEC (enteropathogenic *E. coli*) and EHEC (Enterohemorrhagic *E. coli*), consist of a 35-kb genetic element known as the 'locus of enterocyte effacement' (LEE) [57]. This locus encodes for 41 different genes, at least 5 of which are T3SS-effectors proteins (Tir, Map, EspF, EspH, and EspZ). Many functions have been suggested for these effectors, including re-organizing the actin filopodia and pedestals (Tir, Map and EspH [58], [59], [60]), altering septin cytoskeleton (EspF [61]), and inhibiting apoptosis (EspZ [62]). All these effectors are located and act in the host-cell cytosol. Moreover, their secretion is vital for the initiation of the *E. coli* infection of enterocytes, and are thus expressed at the early stages of the infection [63].

#### Escherichia coli- *selection for CD8+ T cell evasion in T3SS-effector*

Figure 3C represents the comparison between the *SIR* score of these effectors proteins and the score of all non-effectors *E. coli* proteins. As expected, for most of *E.coli* strains tested, T3SS effectors have lower epitope densities than 1 (T test P-value<1.e-3), than other proteins from the same bacterium (ANOVA P-value 0.00248). However, the variance in the *E.Coli* epitope densities among different *E.Coli* strains is very large. The typical EPEC differs significantly at the genetic level from the atypical EPEC and EHEC. In the typical EPEC strains (O127:H6, O103:H2, O111:H-), the *SIR* score of effectors was not significantly lower than both their scrambled sequences and non-effectors proteins (data not shown). These proteins have very high epitope densities in two frequent alleles, A*0201 and B*4001 (*SIR* scores of 1.8 and 3.5 compared with neutral *SIR* scores of 0.8 and 0.7, respectively). Since these alleles have high frequency among the Caucasian population (accumulated frequency of 15%), their donation to the averaged *SIR* score is very high. We have no clear explanation for this observation. However, EHEC O157:H7, the most important cause of severe diseases in the Western world [64] , and its closely related atypical strain O55:H7, have the lowest *SIR* scores, suggesting a strong selection pressure on specific strains. Given this high heterogeneity, we cannot clearly prove selection in *E.Coli* in general.

## T3SS-effectors epitopes have a much lower affinity than other epitopes in bacteria

The absolute number of epitopes or their density might not give the full picture regarding to escape mutations. Such mutations could affect, for example, the quality of the epitopes. We have thus checked if the epitopes still present on T3SS effectors have an affinity similar to epitopes from other proteins. In order for peptides to be presented on MHC-I molecules, they have to pass three stages: Proteasomal cleavage, TAP translocation, and MHC-I binding.

We computed the probability to pass these three stages using proteasomal cleavage, TAP binding and MHC-I binding algorithms. An epitope was defined as a peptide with a supra-threshold score at each stage.

**Figure 4 pcbi-1002220-g004:**
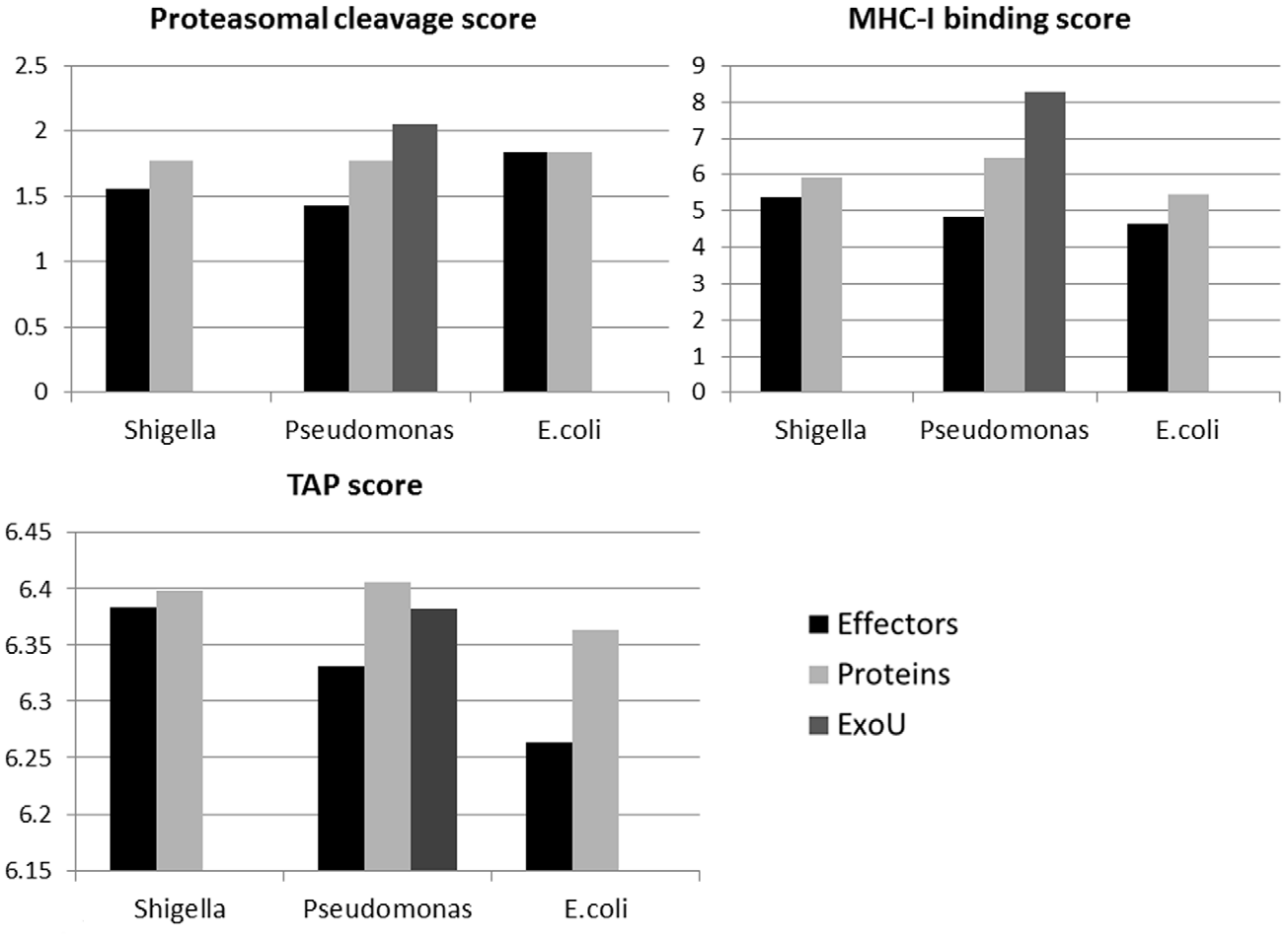
Average scores of epitope for all steps of epitope presentation (proteasomal cleavage, TAP binding and MHC-I binding) in *Shigella flexneri*, *Pseudomonas aeruginosa* and *Escherichia coli*. The y axis values represent the score in each step. *P. aeruginosa* effector, ExoU, being a unique necrosis effector was examined separately. In all bacteria tested, the average scores of epitopes in effectors in all stages are lower than the scores of epitopes in randomly selected proteins (with one exception of proteasomal cleavage of *E.coli*). ExoU epitopes had higher proteasomal cleavage and MHC-I binding scores in comparison to these scores of randomly selected proteins. Note that TAP is the less limiting factor in epitope processing. All differences (except proteasomal cleavage of *E.coli* with P-value = 0.388) are significant with 1.e-10<P-value<0.06.

The averaged proteasomal cleavage, MHC-I binding and TAP binding scores of epitopes derived from random bacterial proteins and from effectors of the three gram-negative bacteria used in this study are represented in Figure 4.

One can clearly see that most effectors have consistently lower scores for cleavage, TAP binding and MHC binding. (T test 1.e-10<P-value<0.06) with two exceptions: ExoU, that, consistent with our previous results, has proteasomal cleavage score and binding score higher than randomly selected proteins, and proteasomal cleavage of *E.coli* where the differences are not significant (T test P-value = 0.388). Since these scores correspond to the probability that a given peptide will be eventually presented at MHC-I molecule, these results highlight again the efforts made by the bacteria to prevent T3SS-effectors recognition by CD8+ T-cells: not only are there less epitopes in T3SS effectors, but the remaining epitopes have lower probabilities of being presented.

## Intracellular bacterial toxins are similar to Gram Negative T3SS effectors in terms of immune-induced evolution

Cytosol localization of bacterial proteins is not unique to T3SS effectors. While Intracellular bacteria are localized within host cells, they usually do not reach the cytosol. Most of the bacteria reside in the phagosome of the host cell. However, some bacterial proteins are exposed to the host cytosol even in Intracellular bacteria. Two examples for such bacteria are *Listeria monocytogenes* and *Mycobacterium tuberculosis*.

## *Listeria monocytogenes*

**Figure 5 pcbi-1002220-g005:**
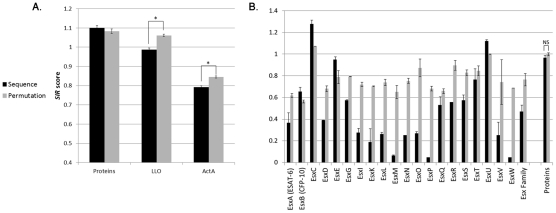
*SIR* score of cytosolic proteins and randomly selected proteins in *Listeria monocytogenes* and *Mycobacterium tuberculosis*. A) Comparison between the average *SIR* score in real sequences and in scrambled sequences in *Listeria monocytogenes*. The cytosolic proteins Listeriolysin O and ActA (second and third columns, respectively) have lower *SIR* scores than randomly selected proteins and than their scrambled sequences (P-value<1.e-12 for both proteins). In randomly selected proteins (first column), the differences between real and scrambled sequences was insignificant (P-value = 0.8652). B) Comparison between the average *SIR* score in real sequences and in scrambled sequences in *Mycobacterium tuberculosis*. The first two columns are the average *SIR* scores of EsxA and EsxB. Columns 3-20 are the scores of ESAT-6 homologues hypothetical proteins. Column 21 is the averaged *SIR* score over all proteins in the Esx family, and the last column is the averaged scores of 400 randomly selected proteins. In EsxA (ESAT-6), as well as in 15 out of 18 ESAT-6 homologues, the average SIR score in the real sequence is lower than the SIR score in randomly selected proteins and their scrambled sequences. In randomly selected proteins, the differences between real and scrambled sequences was insignificant (P-value = 0.2212). These results argue that the hypothetical ESAT-6 homologues - like ESAT-6 itself – might be localized in the host cytosol. *NS-not significant. All other differences are significant with P-value<0.05.

*L. monocytogenes* can escape from the phagosome and remain in the cytosol. This escape occurs through the secretion of pore forming toxin- listeriolysin O (LLO) [65] that degrades the phagosomal membrane. LLO is a member of cholesterol-dependent cytolysins (CDCs) – a large group of pore-forming toxins that depends on membrane cholesterol for their activity. This group consists of about 20 members (for a review see [66]), each produced as a soluble monomeric protein that, in most cases, is secreted by a type II secretion system. LLO is known to reside in the cytosol. However, cytosolic LLO is much less active as a pore-forming toxin. Instead, it is highly degraded due to a PEST-like sequence that promotes its targeting to proteasomal cleavage, preventing the pore forming in the cell membrane and the sequential lysis of its host cell [67]. While in the cytosol, *L.monocytogenes* secretes another protein, ActA that is used for actin polymerization and horizontal movement within the intestinal epithelial layer [68]. As expected from the results in the previous sections, both LLO and ActA have a lower SIR score than 1 (T test P-value<7.6e-12) and both their scrambled versions (ANOVA P-value = 7.e-7), and randomly selected proteins (T test P value<1.e-12 for LLO and ActA, separately, and ANOVA P-value<1.e-12 for both proteins together). This suggests an immune escape strategy of *L. monocytogenes* as in gram negative bacteria. As in all previous cases, the average over randomly selected proteins of *L.monocytogenes* does not show such a decrease in the epitope density (Figure 5A).

## *Mycobacterium tuberculosis* cytosolic proteins

*Mycobacterium tuberculosis* (TB) [61] resides in the phagosome of lung macrophages. In MB, the ESAT-6 (esxA) and CFP10 (esxB) proteins are secreted into the host cell and were proved to reach the cytosol [22]. The access of these ESX-1 proteins to the cytosol might be achieved either by the TB escape from the phagosome or translocation of these proteins to the cytosol through sec61, or alternatively directly by ESX-1. Consistent with these last two options, these proteins were shown to induce CD8+ T-cell response regardless of the escape of the bacteria from the cytosol [22]. Besides these two proteins, there is a group of at least 18 ESAT-6 homologues. Very little is known about these proteins, but they show homology to the ESAT-6 protein and are thus suspected to be secreted by the same system [69].

## *Mycobacterium tuberculosis SIR* scores

We tested both ESAT-6/CFP10 proteins and ESAT-6 homologues for their *SIR* score. Overall *SIR* scores of ESAT-6 family proteins are lower than 1 (T test p<1.e-15) and than their scrambled versions (ANOVA P-value = 3.5e-9) as well as in comparison to randomly selected *tuberculosis* proteins (ANOVA P-value<1.e-13). Moreover, when checking each protein separately , ESAT-6, as well as 15 out of 18 of its homologues have shown to have lower *SIR* scores than both randomly selected proteins and their own scrambles sequences (T-test P-value<0.05). CFP-10 and the ESAT-6 homologues esxC, esxE and esxU have higher *SIR* scores than their neutral *SIR* scores (T-test P-value<0.05) (Figure 5B). Two of the above proteins, esxE and esxU, are regulated by the same protein, sigM, a member of the extracytoplasmic function subfamily of alternative sigma factors, and were suggested to function in host modulation at later stages of infection but seemed to have no importance in the pathogenesis of acute infection [70]. One could assume that the consistent low *SIR* score in most of this family members' is due to sequence similarity. We have calculated the edit distance between members of the ESAT-6 family (divided by the length of the longer among the compared proteins). As shown in Figure S2, most of ESAT-6 family members are very different from each other in their sequences. This result suggests that the selection for immune evasion has occurred in each protein separately. One can thus summarize that CTL epitopes modulation is a mechanism common to practically all cytosolic bacterial proteins.

## The interesting case of ExoU – an indirect killer

In contrast with all other confirmed effectors, the *SIR* score of *P. aeruginosa's* ExoU was significantly higher than its neutral *SIR* score (T test, p <1e-9). Moreover, the candidate epitopes of ExoU have a higher proteasomal cleavage and MHC-I binding scores than other effectors or non-effector proteins (Figure 4). Thus not only is ExoU not trying to hide, it seems it is making every possible effort to expose itself. Taking into account that ExoU is secreted by cytopathic strains of *P. aeruginosa* and is known to induce rapid cell death in host-cells, we propose that these *P. aeruginosa* strains may use the host immune system to induce cell death. Since the goal of ExoU expression is to kill the cell, having the cell recognized by CTLs may be the easiest way to obtain this goal. The utilization of the host's immune response by bacteria was suggested recently by Gagneux et al [71]. In their study on TB, they detected hyper-conserved epitopes in MTBC (*Mycobacterium tuberculosis* complex) proteins, and suggested that the bacteria benefit from T-cell recognition. Similarly, the extremely high epitope density found in the ExoU protein suggests that over-presentation of this protein acts to induce CD8+ T-cell response in the host-cell by the cytopathic strains of *P. aeruginosa* as part of their mechanism to induce cell death. We are now looking for similar effects in viruses.

## Validation with other algorithms

In this study we have used the *SIR* formalism as used in our previous studies. While this formalism was validated for some alleles, its MHC-binding algorithm (BIMAS) is relatively old and new algorithms have been introduced since then for some alleles. In order to test that our results are not an artifact of the algorithms used, we have tested the validity of our results using two other algorithms: MLVO and NetMHC (see method section for a detailed description of these algorithms).

**Figure 6 pcbi-1002220-g006:**
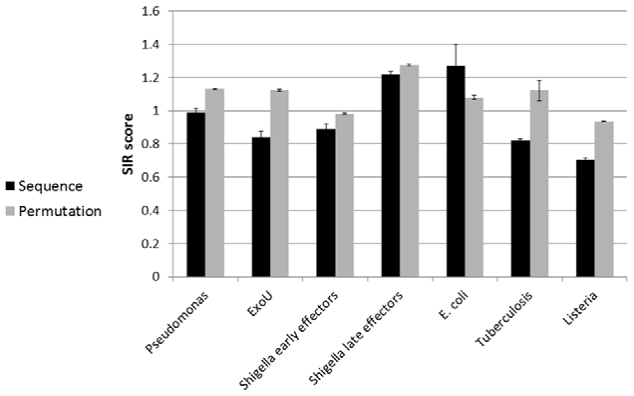
Validation of the results with MLVO algorithm. In *Pseudomonas*, *Shigella*, *Micobacterium* and *Listeria*, the cytosolic proteins have lower *SIR* scores than expected, consistent with our previous results. However, the *E.coli* effectors had higher *SIR* scores than expected from their sequence and ExoU have shown a lower *SIR* score than expected, in contrast with the results using BIMAS. (P-value<0.08 for late *Shigella* proteins and P-value<0.04 for other proteins).

When using the MLVO, the results were similar to the traditional *SIR* score (based on BIMAS) results, and were often more significant. For most bacteria tested, all effectors were shown to have a lower *SIR* score than expected from their sequence. The results were significant for most groups of proteins (Figure 6, ANOVA p <0.03). The exception were again the *E.Coli* that showed a high variability among strains and proteins, and late effectors of *Shigella* in which no significant differences were shown (ANOVA P-value>0.5 for both *E.coli* and late *Shigella* effectors). The main difference between the MLVO and BIMAS results was that in the MLVO formalism, the *SIR* score of ExoU was lower than its scrambled versions (T test P-value<0.04). Although the accuracy of MLVO is better than most other algorithms for the vast majority of alleles, this algorithm was not systematically tested on other organisms. We thus use the MLVO results at this stage only as a validation of the *SIR* results.

**Figure 7 pcbi-1002220-g007:**
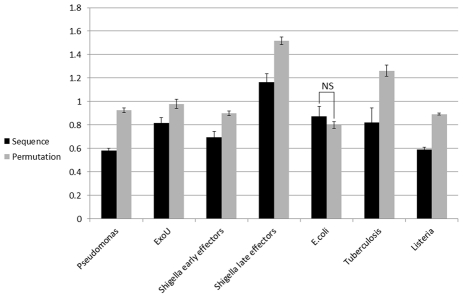
Validation of the results with NetMHC algorithm. In *Pseudomonas*, *Shigella*, *Micobacterium* and *Listeria*, the cytosolic proteins have lower *SIR* scores than expected, consistent with our previous results (P value<0.02). However, *E.coli* effectors showed no significant differences between the real and neutral *SIR* scores and ExoU have lower *SIR* score than expected (P value = 0.012), in contrast with the results using BIMAS.

To further validate the results, we have repeated the analysis using NetMHC. In most bacteria tested (again, with the exception of *E.Coli* and late effectors of *Shigella* in which the differences was not significant (ANOVA P-value>0.58 and 0.064, respectively)), the *SIR* score predicted by the NetMHC of cytosolic proteins was lower than their neutral *SIR* score (Figure 7, ANOVA P-value <5.e-3). Consistent with MLVO but in contrast with BIMAS formalism, ExoU score was lower than expected (T-test P-value = 0.012).

Taken together, in most cases our results using BIMAS algorithm were in agreement with the results of MLVO and NetMHC algorithms, and that the observed reduction in the number of epitopes is not an artifact of a specific algorithm. A summary of the significance of the results in all three algorithms are presented in Supplementary Material (Table S3).

## Discussion

We have performed a broad analysis of immune-induced selection of CD8+ T-cells escape mutations in cytosolic bacterial proteins. While in general CD8+ T-cell response induces very weak selection, if at all, on bacterial proteins, a strong selection was observed on the T3SS-effectors group of gram-negative bacteria and probably on cytosolic bacterial proteins in general. Furthermore, the strength of the selection on the effectors depends on their time of expression as can be seen in the case of *S.flexneri* where the early set of effectors was selected more strongly than the late set. These results are in good agreement with our previous studies on herpesviruses [43], HIV [24] and viruses in general [25], showing that proteins expressed in phases critical to the fate of infection (e.g., early lytic and latent) evaded immune detection more than others.

In order to validate these results, we have repeated the analysis using a recently developed algorithm (the MLVO), as well as the more classical NetMHC with similar results for the vast majority of the proteins.

An intriguing possibility is that the direction of selection depends on the function of the effectors. This was demonstrated by the *P. aeruginosa* cell death mediated effector ExoU that has evolved to have more epitopes, and thus, might induce CTL response. The involvement of ExoU in inducing CTL response is in good agreement with studies of corneal infection by the *P. aeruginosa* strain which was shown to be dependent on ExoU secretion [72]. Barrett et al. [73] have shown that mouse strains favoring development of a Th1-type response are susceptible to corneal infection, suggesting the involvement of CTL response in this infection. Note that this result was not observed using MLVO, and is thus left as a hypothesis to be checked further.

In *E.Coli*, a very high variability in the epitope density of proteins and strains has been observed, as well as a large difference between the epitope densities in different HLA alleles. We currently have no clear explanation for this variability, except perhaps for a specific adaption of EPEC and EHEC to different populations and thus different epitope densities distributions among HLA alleles. Thus, in contrast with all other bacteria tested here, we cannot safely claim that *E.Coli* effectors proteins have evolved to avoid detection. Further research is needed to understand the peculiar differences between *E.Coli* strains.

**Figure 8:**
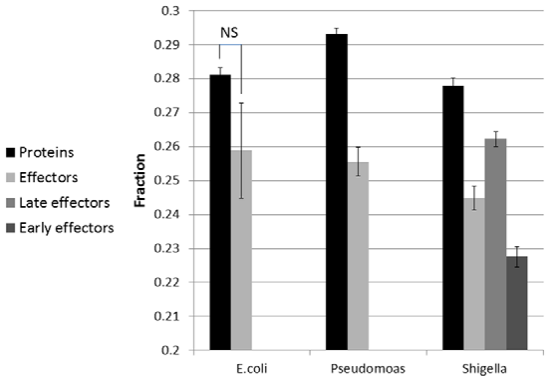
Fraction of peptides derived from proteasomal cleavage in *Escherichia coli*, *Pseudomonas aeruginosa* and *Shigella flexneri*. T3SS effectors have less possible cleavage- derived nine-mers than other proteins. Moreover, in *S. flexneri*, among effectors, early secreted effectors have less cleavage-derived nine-mers than the late secreted ones. *P-value<1e-4 for *P.aeruginosa* and late effectors of *S. flexneri*, and P-value<1e-44 for early effectors of *S. flexneri*. In *E.coli*, the differences in fraction of cleavage derived peptides between effectors and other proteins was not significant (P-value = 0.1).

Compared with viruses, bacteria have a relatively low mutation rate of approximately 1.e-8 (as compared with approximately 1.e-5 in viruses). Considering the lack of species specificity and the horizontal transfer of many genes, including the members of type III secretion system, bacteria are much less genetically flexible, and therefore, epitope density within a protein might be influenced not only by the immune-induced selection but also by the time when the horizontal transfer took place and the variety of species infected by the bacteria, forcing them to adapt to different HLA alleles and other species-specific constraints. A way to maximize the evolutionary conservation of epitopes (or the lack of epitopes) is to directly affect the cleavage mechanism that is common to most mammals. Indeed, when computing the proteasomal cleavage ratio (number of nine-mers that are the results of proteasomal cleavage divided by the total number of nine-mers), effector proteins had a lower ratio than other proteins in all bacteria. These results were significant for *P. aeruginosa* effectors and late and early *S. flexneri* effectors (T-test, P-values = 5.7e-5, 4.3e-5 and 6.6e-45, respectively), and insignificant for *E. coli* effectors (p = 0.1) (Figure 8).

The current analysis shows that an important part of the immune response against bacteria may be the CTL response against cytosolic bacterial proteins. This response may be a key element in the development of future anti-bacterial therapies.

## Methods

### Bacterial sequences

*Pseudomonas aeruginosa, Escherichia coli, Shigella flexneri, Listeria monocytogenes* and *Mycobacterium tuberculosis* gene sequences were used for this analysis. The sequences were obtained from the NCBI (http://www.ncbi.nlm.nih.gov/) database. All sequences are available in the Supplementary Material. For *P. aeruginosa*, we used 16 ExoU sequences and 18 sequences of the 3 other effectors. For *S. flexneri*, we used 75 early effectors and 30 late effectors sequences. For *E. coli*, we used 38 effectors sequences (11 Tir, 4 EspF, 4 EspH, 14 EspZ and 5 Map). For *L. monocytogenes*, we used 107 listeriolysin sequences and 483 ActA sequences. For *M. tuberculosis*, we used 62 Esat-6 proteins sequences. For all bacteria, we took 400 sequences of random non-effectors proteins. For each protein sequence, we produced 50 scrambled sequences as a reference.

### *SIR* score

We have analyzed the ratio between the number of epitopes presented in bacterial proteins and their random counterpart. This ratio was defined as the Size of Immune Repertoire (*SIR*) score. The epitope number was computed using three algorithms: a proteasomal cleavage algorithm [39], a TAP binding algorithms developed by Peters et al. [40] and the BIMAS MHC binding [74] algorithms. We have computed epitopes for the 33 most common HLA alleles and weighted the results according to the allele frequency in the global human population (Figure 1). The algorithms' quality was systematically validated vs. epitope databases and was found to induce low FP and FN error rates. The computation of the SIR scores can be performed through our web-server at http://peptibase.cs.biu.ac.il/index.html.

The comparison between effectors and their scrambled sequences, as presented in Figures 3–8, was done on the average of the entire group of proteins. We have also tested the possibility of first averaging each protein separately and then to average the results over all proteins, as we have previously done for some viral proteins [25], [43], [44], [75]. There is no major difference between the results in the two approaches. The results using the latter approach are represented in the Supplementary Material (Figure S1).

### Cleavage score

Given a peptide with N- and C-terminal flanking regions *FN* and *FC* and residues *P*1, .*Pi*, . . *Pn*, where *Pi* represents any residue 1, and *n* represents C and N positions, the following score was defined:
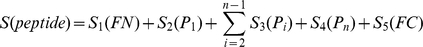
A peptide with a high score, *S*, has a high probability of being produced, while a low score corresponds to a low probability of production. The appropriate values for 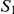 to 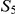 were learned using a simulated annealing process [76]. The algorithm was validated to give a rate of false positives of less than 16% and a rate of false negatives of less than 10% [39].

### TAP binding frequency

The probability that a peptide binds the transporter associated with antigen processing (TAP) machinery is mainly a function of the residues at the first three N-terminal and the last C-terminal positions. Moreover, this probability can be estimated through a linear combination of the binding energies of the residues. Multiple algorithms for TAP binding frequency were checked. The score computed by Peters et al. [77] gave the best differentiation between presented and random peptides [46].

### MHC binding motifs

Each protein was divided into all possible nine-mers by using a sliding window (e.g., a 300-amino-acid protein was divided into 292 nine-mers, positions 1 to 9, positions 2 to 10, and so on). For each nine-mer, we computed the MHC binding energies of 31 different class I human leukocyte antigen (HLA) molecules, most of them HLA-A and HLA-B. The affinity of a candidate peptide for each HLA molecule was estimated using the BIMAS software and the binding coefficients predicted by Parker ([78]; http://www-bimas.cit.nih.gov/molbio/hla_bind/).

These matrices estimate the contribution of each amino acid at each position to the total binding strength. While many more modern algorithms exist for MHC binding prediction, we have previously found the BIMAS algorithm to provide trustworthy results in most highly frequents alleles that compose the bulk of the score analyzed here [24], [33], [43].

### Multi-Label Vector Optimization (MLVO)

The MLVO algorithm [79] for MHC and TAP binding prediction finds a classifier (w) using three label types that are combined into a single constrained optimization problem. The method finds the optimal combination of binary classification of peptides known to bind or not to bind the MHC/TAP molecule, a linear regression based on the measured affinities of peptides with a known IC50 or EC50 binding concentrations and a guess (often based on information on similar alleles). In the current analysis, we have used the MLVO algorithm for MHC binding [79], as well as for TAP binding. The MHC binding accuracy of the vast majority of MHC-I alleles in the MLVO is over 0.95 (with AUC of over 0.98) [79]. As in all other cases, the SIR results presented are a weighted average over alleles of the ratio between the computed epitope density and the one expected in a random sequence.

### NetMHC

The NetMHC algorithm uses an artificial neural network (ANN) based method for MHC binding prediction [80]. The ANN is trained by eluted MHC ligands for which binding affinity data is measured. We define an epitope as a peptide that exceeds the threshold of 500 nM ('weak binder'), and calculated the *SIR* score accordingly. In order to compare the NetMHC results to the BIMAS and MLVO results, we applied the Ginodi cleavage algorithm [39] and the Peters TAP binding score [40] . Only peptides having a supra-threshold score were tested for MHC binding. Again, the *SIR* results presented are a weighted average over alleles of the ration between the computed epitope density and the one expected in a random sequence.

### Thresholds

The different epitope prediction algorithms provide a binding score. In order to produce an epitope list, a cutoff should be applied to these scores. There are two possibilities to use thresholds for the definition of epitopes: a single affinity threshold for all alleles, or an allele dependent threshold. The first attitude is based on the need to bind the MHC molecule for a long enough period to activate T cells. The second attitude is based on the competition for the presentation on a limited number of MHC molecules. For example, an allele such as B*2705 is expected to present a very large number of epitopes from self proteins [81]. Thus a viral protein with a large number of epitopes would have to compete with a similarly high number of epitopes in human proteins. We here use the second option where we have computed an allele specific presentation threshold value that limits the number of predicted presented epitopes from a random sequence (Supplementary Material, Table S1). While this may lead to the exclusion of some real viral epitopes, it should not affect the ratio between the number of computed epitopes in real and scrambled sequences. Cutoffs for all alleles can be found in the Supplementary Material (Table S1).

### Statistical analysis

The *SIR* score of various populations was compared to the expected score. A two way nested ANOVA was used to compare the *SIR* scores of bacterial proteins in real vs. scrambled sequences, as well as the SIR score of effector vs. other proteins in bacteria. The ANOVA analysis was performed using two layers of variables: the main group -effector/non-effector or real/scrambled and the second, nested within the first is the protein identity.

A two way T-test with unknown and unequal variance was used in cases where no layers has to be considered (comparison *SIR* score of each protein groups to 1, and comparison of the averaged proteasomal cleavage, tap binding and MHC-I binding scores of epitopes in effectors and non-effector proteins).

### Epitope computation

We have designed a CD8+ T cell epitope SQL based library webserver: http://peptibase.cs.biu.ac.il. This website provides detailed CD8+ T cell epitope libraries for the human and mouse genomes as well as for most fully sequenced viruses. It also allows users to upload a file and produce an epitope library. All bacterial proteins in this study were analyzed for their epitope using this webserver.

## Supporting Information

Figure S1 *SIR* score of effector groups, averaged each protein by itself.
(TIF)

Figure S2 Similarity among ESAT-6 like family proteins. Similarities are represented by the edit distance divided by the length of the longer protein among the two proteins that were compared.
(TIF)

Table S1 MHC-I allleles used in the analysis. The first column describes the allele frequency in Caucasian population (http://www.ebi.ac.uk/imgt/hla/). The second column describes the presentage of random epitopes that bind to the allele, and the third column describes the cutoff used by the algorithm to classify binders/non-binders.
(TIF)

Table S2 *SIR* score and neutral *SIR* score for each protein in the study.
(TIF)

Table S3 Comparison between the results of BIMAS, MLVO and NetMHC algorithms and their significance. H/L- *SIR* score is higher/lower than the neutral *SIR* score. NS-not significant.
(TIF)
