# Supplementary figures and images for: Bacteria Modulate the CD8+ T Cell Epitope Repertoire of Host Cytosol-Exposed Proteins to Manipulate the Host Immune Response
Source: PLoS Comput Biol. 2011 Oct 13;7(10):e1002220. doi: 10.1371/journal.pcbi.1002220 (PMC3192822; doi:10.1371/journal.pcbi.1002220)

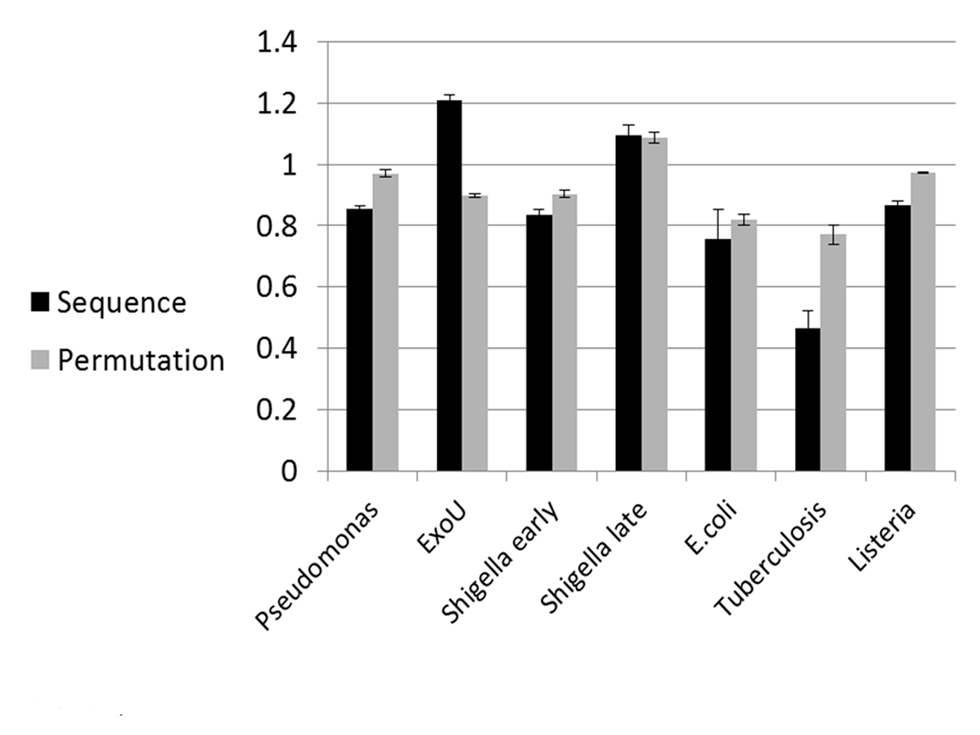

Supplement: Figure S1 — SIR score of effector groups, averaged each protein by itself. (TIF) [file pcbi.1002220.s001.tif]

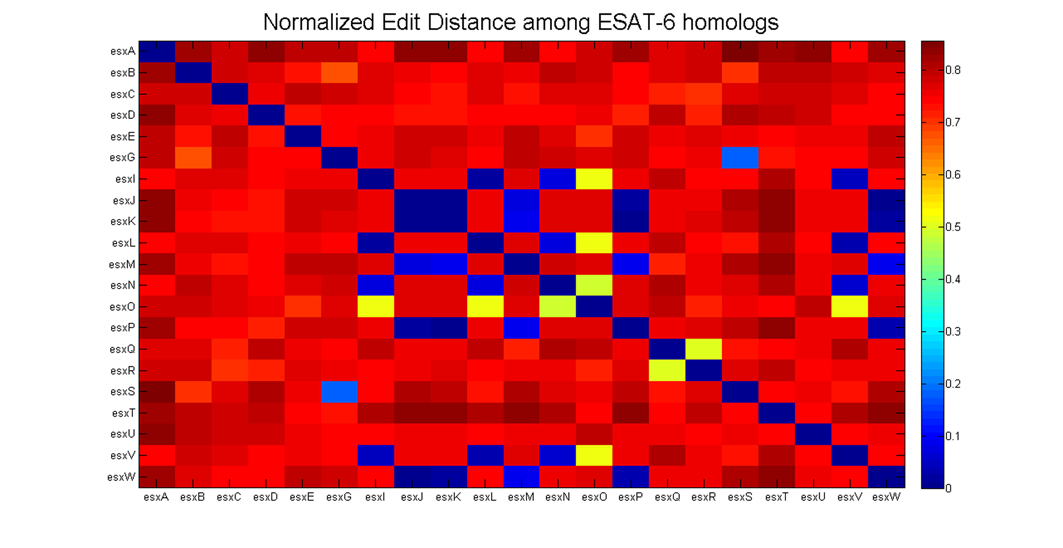

Supplement: Figure S2 — Similarity among ESAT-6 like family proteins. Similarities are represented by the edit distance divided by the length of the longer protein among the two proteins that were compared. (TIF) [file pcbi.1002220.s002.tif]

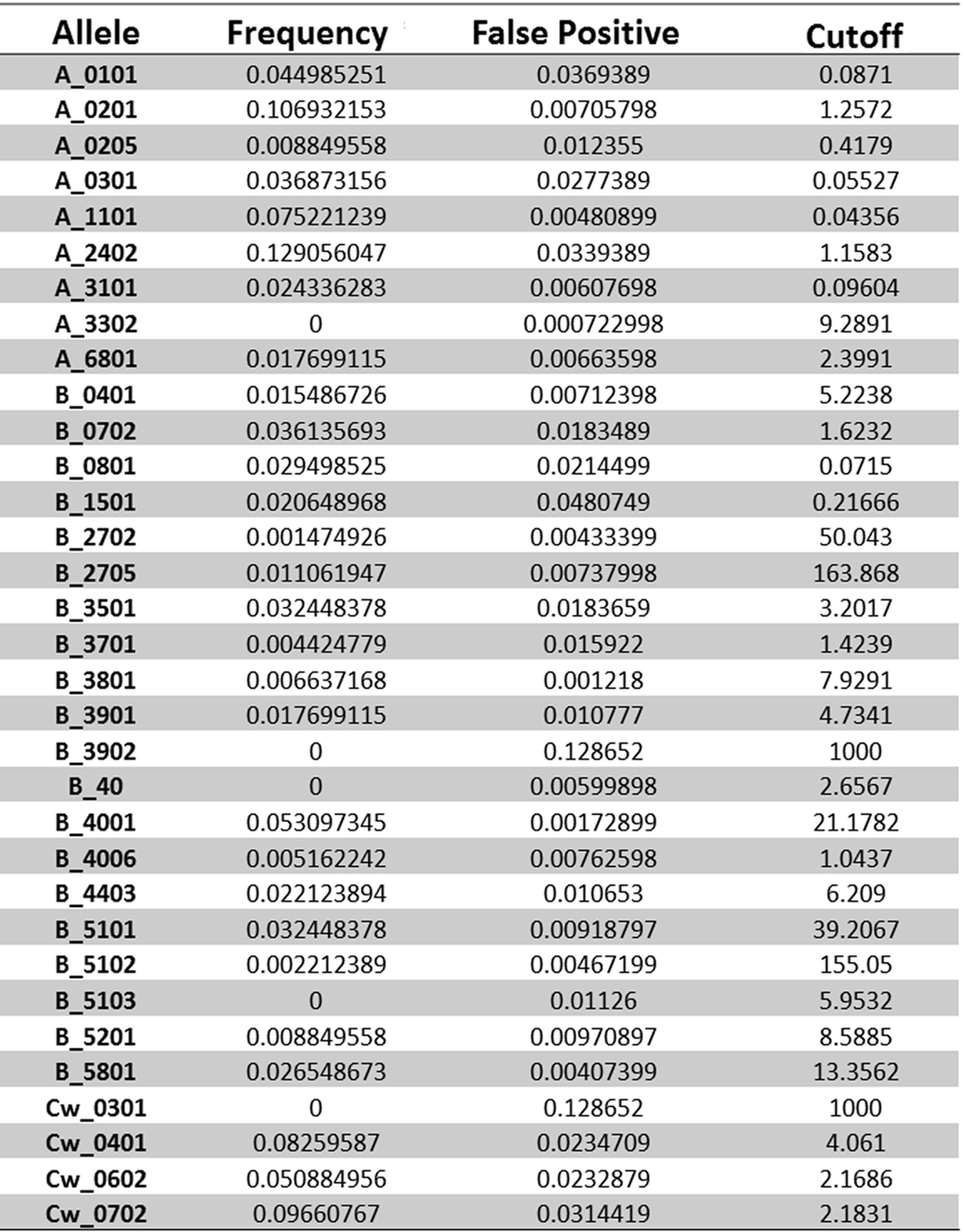

Supplement: Table S1 — MHC-I allleles used in the analysis. The first column describes the allele frequency in Caucasian population (http://www.ebi.ac.uk/imgt/hla/). The second column describes the presentage of random epitopes that bind to the allele, and the third column describes the cutoff used by the algorithm to classify binders/non-binders. (TIF) [file pcbi.1002220.s003.tif]

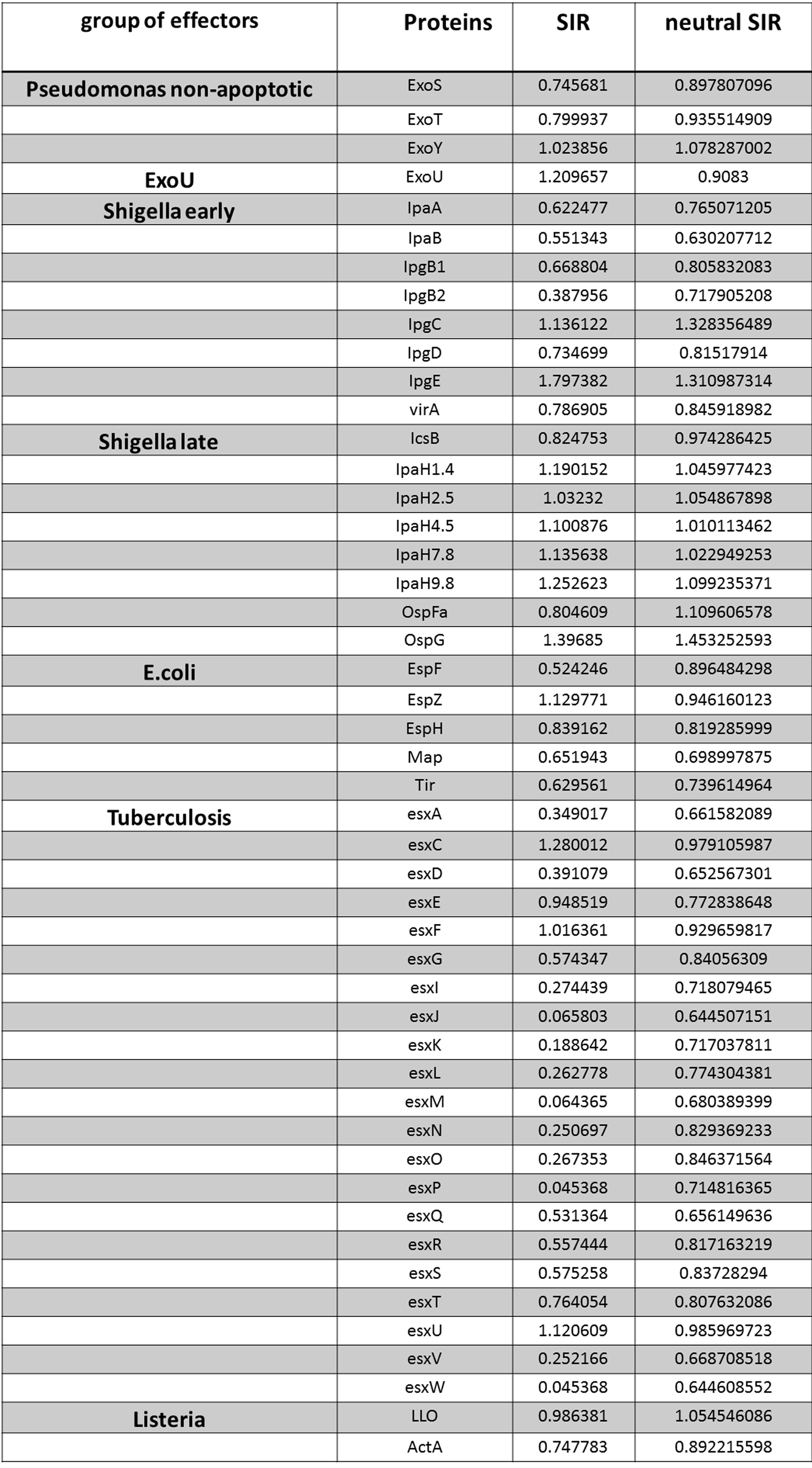

Supplement: Table S2 — SIR score and neutral SIR score for each protein in the study. (TIF) [file pcbi.1002220.s004.tif]

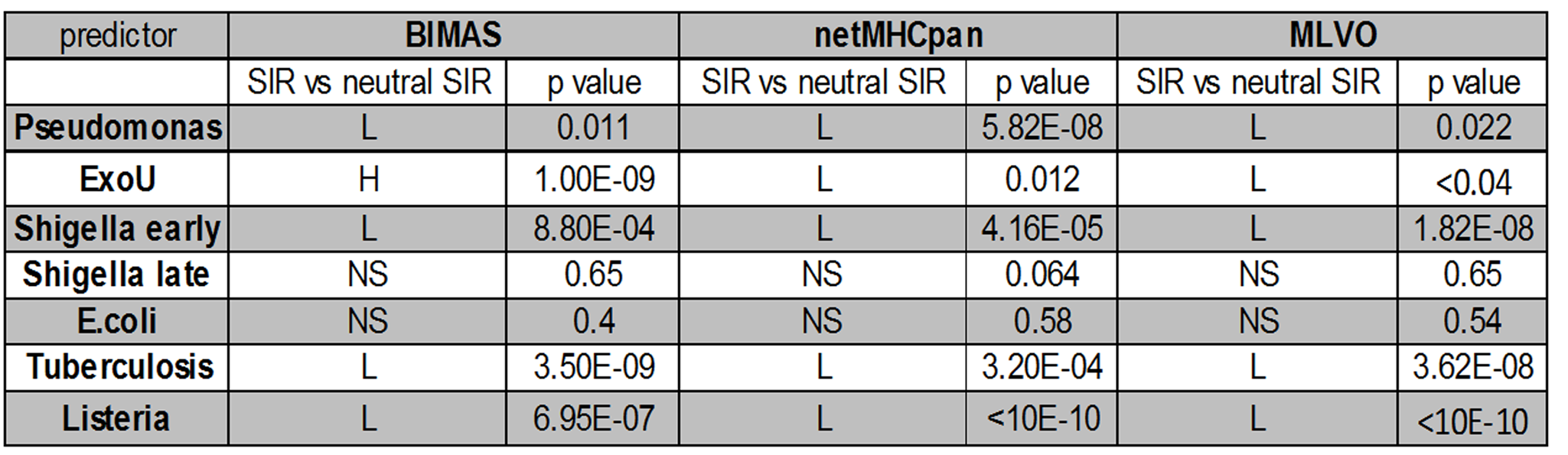

Supplement: Table S3 — Comparison between the results of BIMAS, MLVO and NetMHC algorithms and their significance. H/L- SIR score is higher/lower than the neutral SIR score. NS-not significant. (TIF) [file pcbi.1002220.s005.tif]
